# Successful closure of a cholecystocolonic fistula due to cholecystitis using a clipping system

**DOI:** 10.1055/a-2462-0618

**Published:** 2024-11-29

**Authors:** Noriyuki Hirakawa, Katsuya Kitamura, Kei Yamamoto, Kenichi Tadokoro, Yasunosuke Akita, Takao Itoi

**Affiliations:** 1 89440 Department of Gastroenterology and Hepatology, Tokyo Medical University Hachioji Medical Center, Tokyo, Japan

Idiopathic biliary fistula is an abnormal connection that spontaneously occurs between the biliary system and surrounding organs 1 . The standard treatment is surgery, including cholecystectomy and fistula closure 2 . However, with aging of the population, some patients are unfit for surgery. Recent reports have described the efficacy of the over-the-scope (OTS) clip system (OTSC; Ovesco Endoscopy AG, Tübingen, Germany) 3 4 5 . We report a case in which the OTS clip system was successfully used to close a cholecystocolonic fistula.

The patient was a 92-year-old man who presented with abdominal pain. Computed tomography revealed diffuse thickening of the gallbladder wall and the presence of gallstones and common bile duct stones ( Fig. 1 ). Endoscopic retrograde cholangiography revealed stones in the common bile duct, which were removed using a basket catheter. A hydrophilic guidewire was then used to probe the cystic duct, and a catheter was placed in the gallbladder. Cholecystography showed multiple stones within the gallbladder and extravasation of contrast medium outside the gallbladder, so gallbladder perforation was suspected ( Fig. 2 ).

**Fig. 1:**
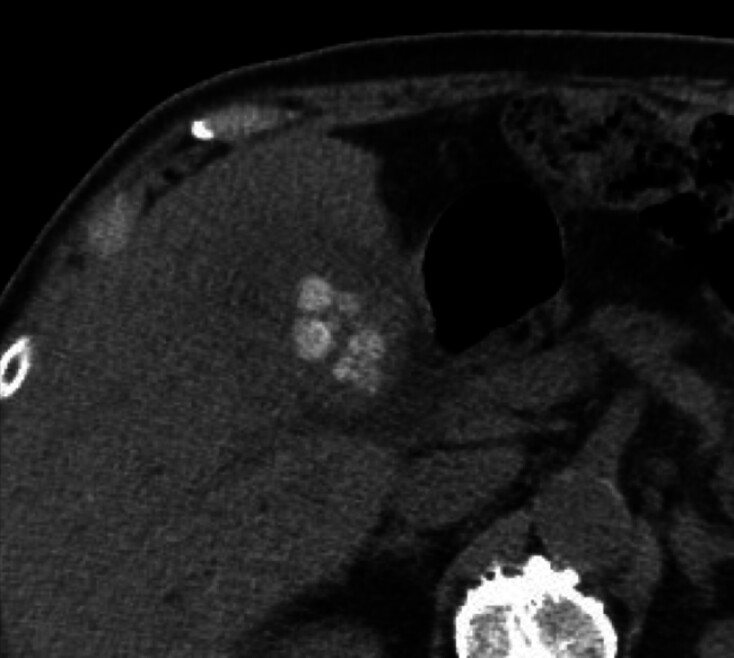
Computed tomography showed gallstones and common bile duct stones.

**Fig. 2:**
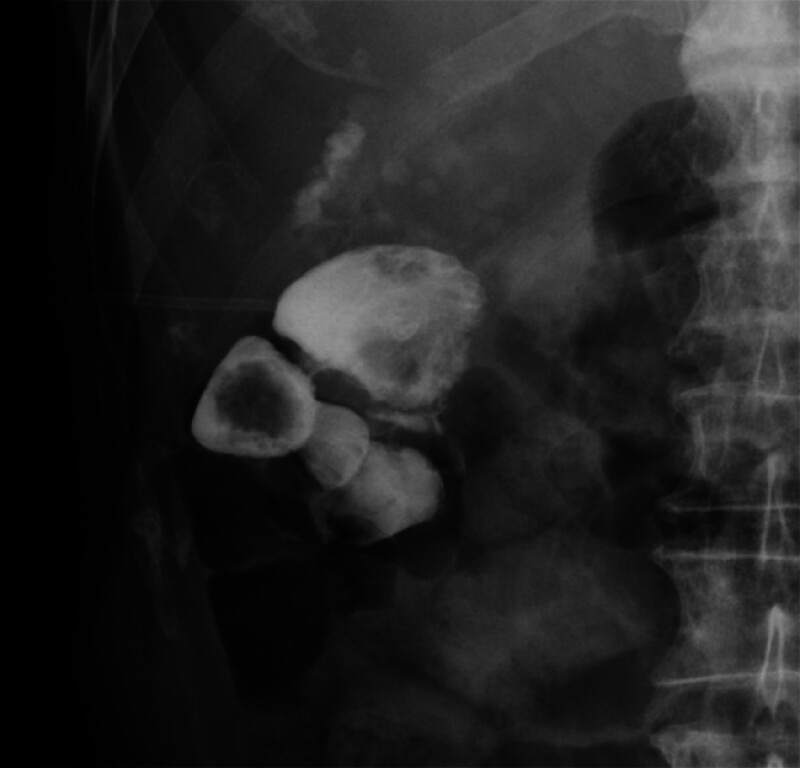
Cholecystography showed leakage into the hepatic flexure of the colon.

To prevent leakage of infected bile into the peritoneal cavity, percutaneous transhepatic gallbladder drainage was performed. Repeat cholecystography revealed leakage into the hepatic flexure of the colon, leading to a diagnosis of cholecystocolonic fistula. Surgery was considered but deemed too invasive given the patient’s age and overall condition. Therefore, endoscopic fistula closure was planned.

A colonoscope was advanced to the hepatic flexure, and the fistula was identified based on cholecystography and endoscopic findings ( Video 1 ). The scope was withdrawn and then reinserted with the cap for the OTS clip system attached ( Fig. 3 ). The colonic mucosa with the fistula was suctioned into the cap. After confirming no leakage into the colon via cholecystography, the fistula was clipped ( Fig. 4 ). After the procedure, follow-up cholecystography confirmed closure of the cholecystocolonic fistula.

**Fig. 3:**
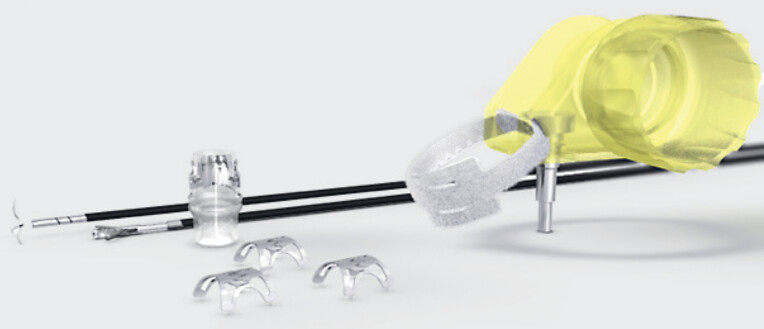
Over-the-scope clip system (Ovesco Endoscopy AG, Tübingen, Germany). Source: Ovesco Endoscopy AG.

**Fig. 4:**
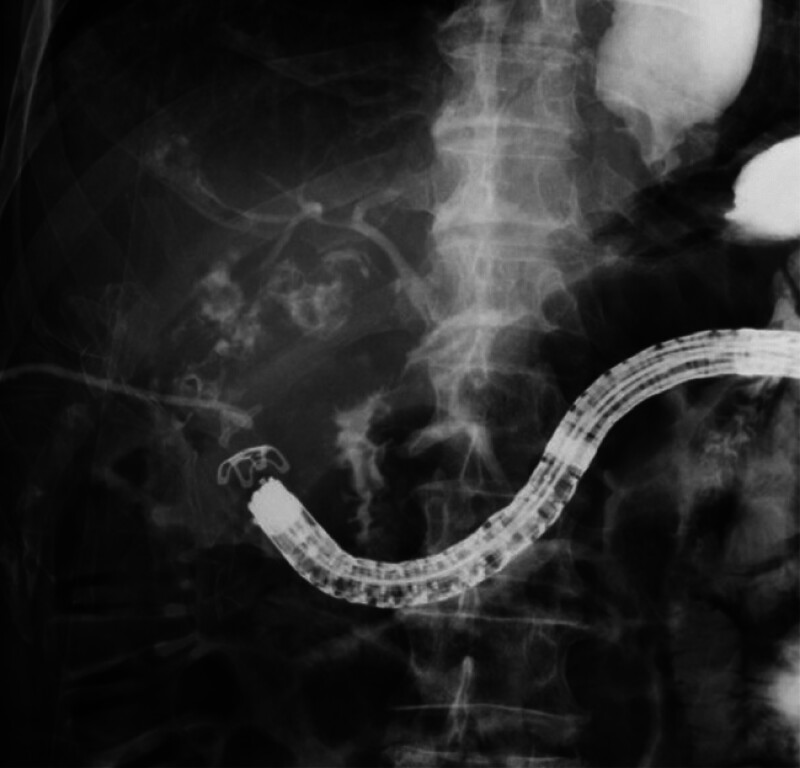
Deployment of the over-the-scope clip.

Successful closure of a cholecystocolonic fistula due to cholecystitis using an over-the-scope clip system. Source for over-the-scope clip system: Ovesco Endoscopy AG.Video 1

Endoscopy_UCTN_Code_TTT_1AO_2AI

## Notes

Endoscopy E-Videos https://eref.thieme.de/e-videos
*E-Videos* is an open access online section of the journal *Endoscopy* , reporting on interesting cases and new techniques in gastroenterological endoscopy. All papers include a high-quality video and are published with a Creative Commons CC-BY license. Endoscopy E-Videos qualify for HINARI discounts and waivers and eligibility is automatically checked during the submission process. We grant 100% waivers to articles whose corresponding authors are based in Group A countries and 50% waivers to those who are based in Group B countries as classified by Research4Life (see: https://www.research4life.org/access/eligibility/ ). This section has its own submission website at https://mc.manuscriptcentral.com/e-videos .
